# Protocol for a cluster randomised controlled trial to determine the effectiveness and cost-effectiveness of independent pharmacist prescribing in care homes: the CHIPPS study

**DOI:** 10.1186/s13063-019-3827-0

**Published:** 2020-01-21

**Authors:** Christine M. Bond, Richard Holland, David P. Alldred, Antony Arthur, Garry Barton, Annie Blyth, James Desborough, Joanna Ford, Christine Handford, Helen Hill, Carmel M. Hughes, Vivienne Maskrey, Kate Massey, Phyo K. Myint, Nigel Norris, Fiona M. Poland, Lee Shepstone, David Turner, Arnold Zermansky, David Wright, On behalf of the CHIPPS Team

**Affiliations:** 1 Institute of Applied Health Sciences, School of Medicine, University of Aberdeen, Foresterhill, Aberdeen, Scotland AB25 2ZD; 2 Leicester Medical School, University of Leicester, Leicester, UK; 3 School of Healthcare, Baines Wing, University of Leeds, Leeds, UK; 4 School of Health Sciences, Faculty of Medicine and Health Sciences, University of East Anglia, Norwich, UK; 5 Norwich Medical School, University of East Anglia, Norwich, UK; 6 School of Pharmacy, University of East Anglia, Norwich, UK; 7 Consultant Geriatrician, Addenbrookes Hospital Cambridge, Cambridge, UK; 8 Norfolk and Suffolk Primary and Community Care Research Office, South Norfolk CCG, Norwich, UK; 9 Athena Care Homes, Unit 2 Rima House, A13 Approach, Ripple Road, Barking, Essex, IG11 0RH UK; 10 School of Pharmacy, Queen’s University Belfast, Belfast, UK; 11 School of Education and Lifelong Learning, University of East Anglia, Norwich, UK; 12 School of Health Sciences, University of East Anglia, Norwich, UK; 13 School of Healthcare, University of Leeds, Leeds, UK; 14 University of East Anglia, Norwich, UK

**Keywords:** Older people, Pharmacist prescribing, Care homes, Polypharmacy, Randomised controlled trial

## Abstract

**Background:**

Prescribing, monitoring and administration of medicines in care homes could be improved. Research has identified the need for one person to assume overall responsibility for the management of medicines within each care home. and shown that a pharmacist independent prescriber service is feasible in this context.

**Aims and objectives:**

To conduct a cluster randomised controlled trial to determine the effectiveness and cost-effectiveness of a pharmacist-independent prescribing service in care homes compared to usual general practitioner (GP)-led care.

**Objectives:**

To perform a definitive randomised controlled trial (RCT) with an internal pilot to determine the intervention’s effectiveness and cost-effectiveness and enable modelling beyond the end of the trial.

**Methods:**

This protocol is for a cluster RCT with a 3-month internal pilot to confirm that recruitment is achievable, and there are no safety concerns. The unit of randomisation is a triad comprising a pharmacist-independent prescriber (PIP) based in a GP practice with sufficient registered patients resident in one or more care homes to allow recruitment of an average of 20 participants. In the intervention group, the PIP will, in collaboration with the GP: assume responsibility for prescribing and managing residents’ medicines including medication review and pharmaceutical care planning; support systematic ordering and administration in the care home, GP practice and supplying pharmacy; train care home and GP practice staff; communicate with GP practice, care home, supplying community pharmacy and study team.

The intervention will last 6 months. The primary outcome will be resident falls at 6 months. Secondary outcomes include resident health-related quality of life, falls at 3 months, medication burden, medication appropriateness, mortality and hospitalisations. A full health economic analysis will be undertaken. The target sample size is 880 residents (440) in each arm) from 44 triads. This number is sufficient to detect a decrease in fall rate from 1.5 per individual to 1.178 (relative reduction of 21%) with 80% power and an ICC of 0.05 or less.

**Discussion:**

Recruitment is on-going and the trial should complete in early 2020. The trial results will have implications for the future management of residents in care homes and the ongoing implementation of independent pharmacist prescribing.

**Trial registration:**

ISRCTN, ID: 17847169. Registered on 15 December 2017.

## Background

In 2012, UK care homes provided personal care and healthcare for almost half a million residents in registered residential or nursing care homes [1]. Care home residents are generally frail, have multiple morbidities and are prescribed a significant number of regular medicines. Furthermore, age-related complex morbidity renders them particularly vulnerable to medication problems and errors. The Care Quality Commission identifies the management of medicines as one area of care in care homes that regularly requires review and continues to fall below the expected standards. The landmark UK-based Care Homes Use of Medicines Study (CHUMS) published in 2009 [2] observed 256 residents in 55 care homes. Almost 70% of residents experienced at least one medication error on any given day. One hundred residents (39.1%) were identified as having one or more prescribing errors including no strength or route of medicine specified (20%), unnecessary medicine prescribed (approximately 25%), and errors in dose or strength (14.4%). Nearly a quarter of residents (57; 22.3%) experienced an administration error such as an omission (11.8%, of all errors). Out of 218 potentially harmful medicines which required biochemical monitoring, 32 (14.7%) had an error.

Many of these medication-related problems were also reported in a systematic review by Alldred et al. [3] which considered interventions to optimise prescribing for older people in care homes. Problems highlighted were prescription of medicines that were no longer indicated, medicines which interacted with concurrent medication, sub-optimal doses, inadequate monitoring and inappropriate duration. The inappropriate prescription of anti-psychotic medicines in care homes, and other medicines for example, benzodiazepines, non-steroidal anti-inflammatory drugs and proton-pump inhibitors, is well documented [4]. This inappropriate prescribing is known to be related to poor quality of life, falls, strokes and increased mortality. In particular, whilst falls are multifactorial in their causation it has been noted that drugs are a modifiable risk factor and periodic drug review should be a component of any falls-reduction programme [5]. Consequently, effective interventions are needed to monitor and discontinue inappropriate therapy.

The CHUMS report [6] proposed that the fundamental failing in care homes was the lack of a healthcare professional with overall continuing responsibility for medicine management, and recommended that a pharmacist should adopt this role working with a lead general practitioner (GP) within each home. The Department of Health (DH) Immediate Action Alert [7] arising from CHUMS required primary care organisations, GPs and community pharmacy contractors to establish effective joint working strategies to address the identified concerns. The resultant predominant model of care is that of a pharmacy team undertaking full medication reviews in care homes on a yearly or biannual basis. Two Cochrane reviews [3, 8] suggest that this model may be sub-optimal and that more effective approaches to medicine optimisation in this population are required.

Changes in United Kingdom (UK) legislation, enabling suitably trained pharmacists to prescribe, provide an opportunity for pharmacist-independent prescribers (PIPs) to assume the proposed central role in the care home environment. Evidence from the UK suggests that PIPs can prescribe safely and provide patient benefit [9]. Recent government initiatives in all devolved UK nations have supported the deployment of pharmacists in both general practices and care homes [10–14]. However, to date, there is no gold-standard randomised controlled trial (RCT) evidence of the clinical or cost-effectiveness of this approach.

The aim of the study described in this protocol is to conduct a cluster RCT, with internal pilot, to compare the clinical and cost-effectiveness of a Care Homes Independent Pharmacist Prescribing Service (CHIPPS) with usual care. This is the final stage of a programme of work, divided into discrete work packages, following the Medical Research Council (MRC) guidance on the development and evaluation of complex interventions [15] which has reviewed the literature to select appropriate outcome measures [16], ascertained the views of stakeholders [17], developed a needs-based PIP training package (publication in preparation) and conducted a non-randomised feasibility study [18].

## Methods

This is a cluster RCT conducted in primary care involving participating GP-PIP-care home triads in four study locations linked geographically to the Universities of East Anglia, Leeds, Aberdeen and Queen’s Belfast, (hereafter referred to by the University identity). A complete list of study sites is available from the Senior Programme Coordinator Mrs. Laura Watts; L.Watts1@uea.ac.uk.

The objectives for the cluster RCT are:

- To use an embedded (internal) pilot study to confirm:
  - ◦ the feasibility of recruiting sufficient GP practices, PIPs, care homes and residents
  - ◦ the availability of data for primary outcome at 3 months
  - ◦ that there are no intervention-related safety concerns

If the pilot is successful, to deliver a full RCT to:

- Describe the clinical effectiveness of the intervention: PIPs assuming responsibility for medicines’ management of elderly residents in care homes
- To estimate the cost-effectiveness of the intervention

### The intervention

The intervention will be delivered by trained PIPs for a period of 6 months. The training programme comprises 2 days of face-to-face instruction, time in practice to develop relationships with the GP and care home staff, and to address any self-assessed competency gaps supported by a mentor, and a formal final sign-off by a GP, who is independent of the research. The development and evaluation of the training programme will be published separately.

The intervention has been tested in a feasibility study [18]. It involves the PIP, in collaboration with the care home resident’s GP, assuming responsibility for managing the medicines of the resident, including:

- Reviewing resident’s medication and developing and implementing a pharmaceutical care plan
- Assuming prescribing responsibilities
- Supporting systematic ordering, prescribing and administration processes with each care home, GP practice and supplying pharmacy where needed
- Providing training in care home and GP practice
- Communicating with GP practice, care home, supplying community pharmacy and study team

Details of the intervention to be delivered by the PIP are in the CHIPPS Service Specification (Additional file 1) which was developed in previous work packages.

The study PIPs will work closely with the care home staff and the resident’s GP, and communicate regularly with both parties. Once residents are recruited, the local researcher will maintain regular contact with the PIP to ensure adherence to study procedures. During the study there will be a check of a random 20% sample of the pharmaceutical care plans and associated resident documents by a study geriatrician, to ensure clinical appropriateness and safety. Additionally, should any problem arise, the geriatrician will discuss this with the Programme (DW, RH) or Trial (CB, RH) Chief Investigator or local Principal Investigator (DA,CB, CH, DW). At the end of the study period the intervention will cease unless the GP practice and care home mutually agree to continue to deliver it outwith the framework of the research programme.

The comparator will be usual GP-led care. Whilst pharmacists may already be providing some services for care homes, these are usually annual or biannual visits and unlike the intensive approach proposed here. At the end of the study period all PIPs in the control practices will be offered access to the study training. Any medical practices which employ pharmacists to provide services to care homes of similar intensity to that which we propose will be excluded.

### Study participants

The inclusion and exclusion criteria for the study participants are:

#### The PIP

*Inclusion criteria:*

- Registered as a PIP with regulating body (GPhC (England and Scotland) or Pharmaceutical Society of Northern Ireland (Northern Ireland))
- Following CHIPPS study training, can demonstrate to their mentor and independent GP assessor competence to deliver the service specification
- Ability to work flexibly and commit a minimum of 16 h a month to deliver the service for 6 months

*Exclusion criteria:*

- Substantive employment with the community pharmacy (branch/store) which supplies medicines to the care home with which the PIP would work, to protect against conflict of interest
- Already providing an intensive service to the care home, e.g. a monthly visit (or more frequently), and provision of intensive medication-focussed services

#### GP practice

*Inclusion criteria:*

- The GP practice must manage sufficient care home residents to support recruitment of the target of approximately 20 eligible participants^1^

*Exclusion criteria:* none

#### Care homes

*Inclusion criteria:*

- Care Quality Commission (CQC) in England, Care Inspectorate in Scotland or Regulation and Quality Improvement in Northern Ireland, registered specialism as caring for adults aged over 65 years
- Primarily caring for residents aged over 65 years
- Associated with a participating GP practice (i.e. one or more residents registered with a participating practice)

*Exclusion criteria:*

- Care homes which receive regular (e.g. a monthly visit or more frequently), from a pharmacist, providing other intensive medication-focussed services
- Care homes which receive regular (e.g. a monthly visit or more frequently), from another healthcare professional, providing other intensive medication-focussed services
- Care homes which are currently under formal investigation with the Care Quality Commission (CQC) in England, Care Inspectorate in Scotland or Regulation and Quality Improvement in Northern Ireland
- Care homes that are participating in any other study likely to affect the outcome of the CHIPPS trial (e.g. falls intervention study, rehydration study, etc.)

#### Care home residents

*Inclusion criteria:*

- Under the care of the participating GP practice
- Aged 65 years or over
- Currently prescribed at least one regular medication
- They or their appropriate representative is/are able to provide informed consent/assent^2^
- Permanently resident in care home (not registered for respite care/temporary resident)

*Exclusion criteria:*

- Currently receiving end-of-life care, (equivalent to yellow (stage C) of the Gold Standards Framework prognostic indicator) [19]
- Have additional limitations on their residence (e.g. held securely)
- Participating in another intervention research study

### Study outcomes

The study outcomes and data sources are summarised below.

*Primary outcome*

- Fall rate per person at 6 months as documented in the care home falls’ record

*Secondary outcomes*

- Proxy resident EQ-5D-5 L (quality of life) at baseline, 3 months and 6 months [20]
- Face-to-face self-reported resident EQ-5D-5 L (for participants with capacity) at baseline, 3 months and 6 months [20]
- Proxy Barthel Index (physical functioning) completed at baseline, and 6 months by identified member of care home staff [21]
- Fall rate per person in the past 3 months at baseline, 3 months and 6 months as documented in care home records
- Health-service utilisation (and associated costs) in the past 3 months at baseline and in the past 6 months at 6 months’ follow-up, collected from care home and GP records
- Mortality
- Change in hospitalisation rate per person (baseline rate defined as 3 months prior to randomisation compared with hospitalisation rate at 6-month follow-up) collected from care home records
- Drug Burden Index (DBI) [22] at baseline and 6 months with medication data collected from GP records
- Cost-effectiveness of the PIP intervention from the perspective of the NHS and care home

In addition, in the internal pilot stage which is now completed, the following data (stop-go criteria) were collected.

- Quantification of interest from medical practices-PIPs-care home(s) to confirm the viability of planned target recruitment numbers and time line
- > 30% of eligible patients have been recruited (from those invited in each home)
- > 80% of data are available at 3 months for falls data
- No significant intervention-related safety concerns

A detailed process evaluation is being conducted following MRC guidance [23] and will be published separately.

### Participant identification and recruitment

Recruitment and consent will be complex due to the need to identify medical practices with a PIP, recruit homes and then residents for each triad. Initially, PIPs and GPs will be recruited concurrently, with the care homes recruited subsequently, followed by the residents. Copies of recruitment documentation to be used in England and Northern Ireland are attached in Additional file 2. Scottish versions required some slight changes in terminology, to accommodate the different regulations for adults with incapacity, and are available on request.

### PIP and GP recruitment

Eligible PIPs in each area will be identified using local networks, and initial informal contact will be followed by formal invitation to PIP and GP practice (letter of invitation, Participant Information Sheet, Consent Form) and consent. PIPs will be recruited, together with the GP practice with whom they should ideally have an already established close working relationship. Basic demographic information about interested GP practices and their linked care home (e.g. the resident mix, home ownership) will be collected to allow purposive sampling if numbers allow. However, if this does not provide sufficient GP practice-PIP pairings, PIPs and GP practices will be approached separately and linked before care homes are approached.

### Care home recruitment

The participating GP practice will approach one (or more, if necessary) of their eligible care homes and invite them to take part in the study. If the care home manager expresses interest, they will be sent a formal invitation pack by the local researcher (including a letter and Information Sheet). If a care home declines participation, the GP will contact another home and invite them to participate. If there are insufficient residents in one home, then up to two further homes can be recruited. Where a home does not wish to participate, and there is no alternative home, a different GP practice in that area will be identified and recruited and the process to recruit the care home(s) will be repeated.

### Resident recruitment

GPs will identify from their lists of registered patients, those resident in the participating care homes taking one or more medications, and screen them against the study inclusion and exclusion criteria. Reasons for any exclusions will be recorded on a standard form collected by the local researcher. Care home managers will hand out invitation packs (invitation letter from GP, Participant Information Sheet (spoken version if necessary) and consent form) directly to potential resident participants. The care home manager will visit each resident after at least 24 h, and obtain verbal consent for the local researcher to be allowed to approach them to discuss participation in the study. For residents who are considered by the manager to lack capacity, packs will be posted to the resident’s next of kin. To minimise selection bias, packs will be distributed in the order of the list of names from the GP.

The local researcher will meet with interested residents, administer the Capacity Assessment for Residents Form (see Additional file 3) and, if appropriate, take fully informed consent. For those without capacity, there are country-specific regulations to adhere to for each of the home nations; these are detailed in Table 1. The approach is in line with recommended practice [27].

**Table 1:** Obtaining third-party consent for residents without capacity in the three devolved home countries

*England and Northern Ireland*	
A letter will be sent to the supporting relative/friend/potential consultee from the GP, enclosing the Information Sheet and Advice Form for signature. The Information Sheet explains the study and asks if, in their opinion, their friend/relative would have wanted to participate, if they had been able to decide this for themselves, and, if they felt that their friend/relative would have participated, would they be willing to act as consultee and give permission on their friend/relative’s behalf? The potential consultee is asked to complete an Advice Form and send it back within 2 weeks. If there is no response within 2 weeks, another similar letter is sent, asking for return of the completed Advice Form within 1 week, stating that, if this not returned the care home will assume that the friend/relative cannot be a consultee and will then identify someone for this role from within the care home; this person will not be the care home manager and will be completely independent of the study. Capacity to consent is described in the Mental Capacity Act 2005 [24] and involves using personal and nominated consultees. The assent process is consistent with Alzheimer Europe Ethics of Dementia Research [25] and has the support of Alzheimer’s UK. In Northern Ireland there is currently no primary legislation on capacity (according to the General Medical Council) and so decisions about medical treatment and care when people lack capacity must be made in accordance with the common law, which requires decisions to be made in a person’s best interests. Therefore, in Northern Ireland, the procedures used in England will be used.	
*Scotland*	
The letter requesting permission, on behalf of the resident, will be sent to the resident’s Welfare Power of Attorney (WPoA), along with the Information Sheet and Consent Form. If there is no response within 2 weeks, another similar letter is sent. If the WPoA returns the signed Consent Form, then the resident will be recruited into the study. If the WPoA does not return the Form, the resident will not be recruited. In Scotland, capacity to consent is described in the Adults with Incapacity Act (Scotland) 2000 [26] and involves a WPoA who is able to give consent.	

If someone loses capacity during the 6 months of the study, they will remain in the study. This is a specific statement on the Consent Form: ‘I agree to continue participating in the study if I lose capacity before the end of the study’. If someone should lose capacity during the study, continued participation will be confirmed with the next of kin following the same procedures as for initial consent.

At each follow up visit, the care home manager will be asked if any participants have re-gained capacity. Should anyone re-gain capacity during the course of the study, and if the resident is willing, their personal consent to continue will be obtained using the template Resident Recovered Capacity documents, and in England it would be with the original Patient Information and Consent. It is made clear in the Participant Information Sheets that if residents decide not to continue, all the information collected so far will remain in the study, but no further information will be collected.

**Fig. 1:**
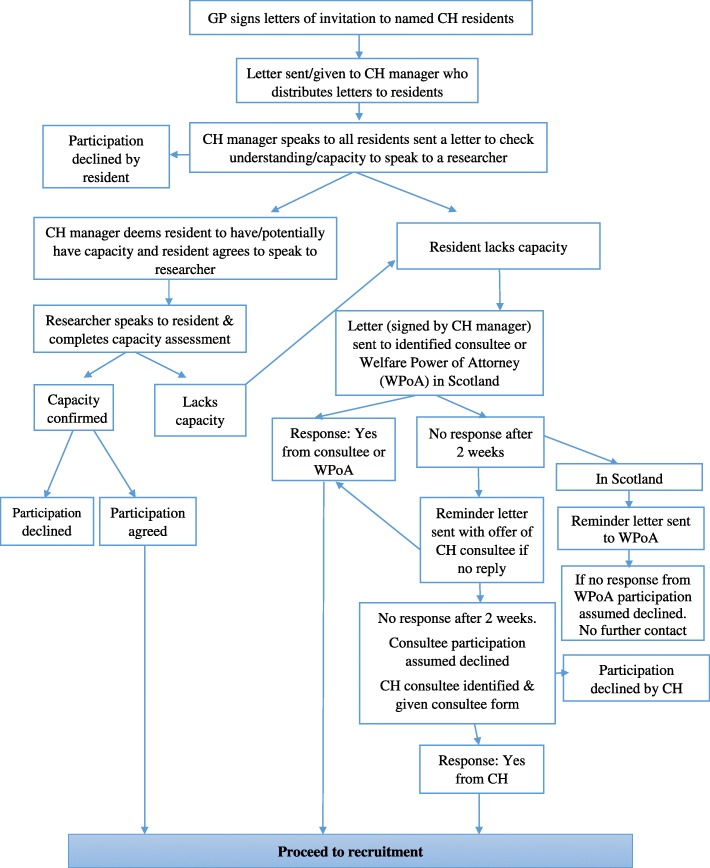
Recruitment flow chart

**Fig. 2:**
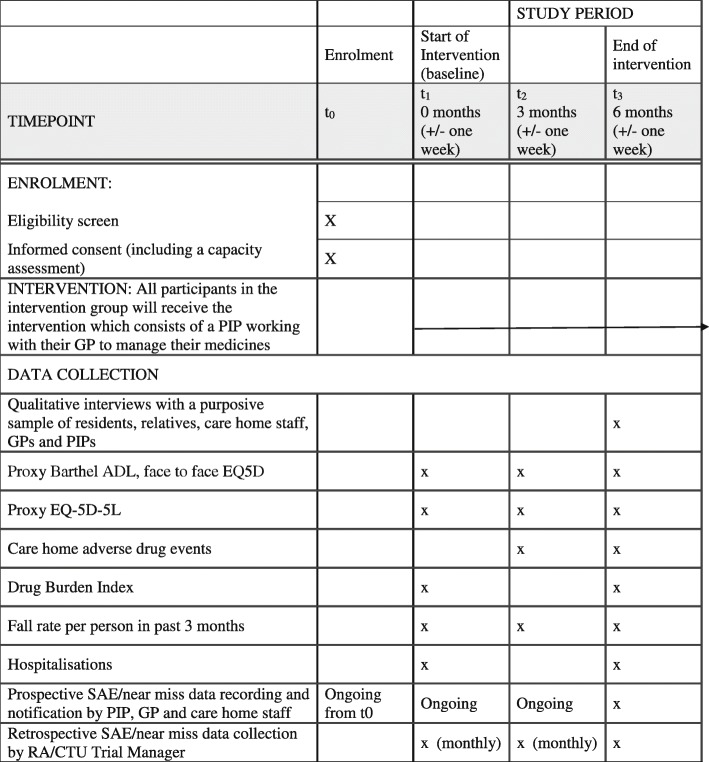
Participant time line

The recruitment flow chart and participant time line are shown in Figs 1 and 2 below.

### Randomisation and blinding

Randomisation will be at practice level rather than the home level in order to minimise contamination which may occur if two homes were in the same practice and one received the intervention whilst the other did not. It is not appropriate to randomise at resident level as the intervention is designed to affect medication-related processes at an institutional (care home), as well as a resident, level and, therefore, control participants would not be immune to its effects.

Blocked randomisation will be undertaken by geographical area, using a web-based electronic randomisation system integrated into the study database. The triads will be informed of their randomisation group by the Senior Programme Coordinator. Local Principal investigators at each geographical site will be informed of the allocations of their triads, (CB, DW, DA, CH) and one of the trial co-CIs (CB) will be informed of all allocations by coded emails. The GP and care homes in each triad are blinded until the care home residents have been recruited. The PIPs are unblinded once randomisation has been completed as the intervention PIPs have to complete training and competency assessment prior to the intervention start. Due to the nature of the intervention, study participants cannot be blinded to the intervention. The local researchers will be blinded until after care home residents have been recruited and baseline data collection has been completed. Should they inadvertently be unblinded they are asked to inform the Senior Programme Coordinator. As the researcher may or may not be correct in suspecting that they know the group allocation their perceived unblinding is not confirmed by the Senior Programme Coordinator until after baseline data have been collected. The potential unblinding is noted on the non-conformance report which is reviewed by the PSC and DMC. The trial statistician is advised of the triads where there is potential unblinding and will assess whether this appears to have resulted in any bias in reporting by comparison with triads where there was no reported unblinding.

### Data collection

Data, as specified earlier, will be collected, by the local researcher, from GP practice and care home paper and/or digital records. Data will be coded and entered into either paper Case Record Forms (CRFs) or electronically using tablets. Data entered on paper records will be subsequently entered into a centrally held Norwich Clinical Trials Unit (NCTU) CHIPPS REDCap [28] database by local researchers. Data collected electronically will be entered into the REDCap database at the time of data collection if there is Internet connectivity, or if working off-line, at the next time the device is synchronised. Data will be protected using established NCTU procedures.

### Data management

Data management is detailed in the Data Management Plan version 1: 21 November.2017. Local research staff will receive training in all aspects of data collection and management. Identification logs, screening logs and enrolment logs will be kept at each of the four University locations in a locked cabinet within a secured room. All data will be handled in accordance with the General Data Protection Regulations 2018. All participants (GPs, care homes and residents) will be given a unique study Participant Identification Number (PIN). Data will be entered under this identification number onto the centrally held database stored on the servers based at NCTU. Access to the database will be controlled with unique usernames and encrypted passwords, and restricted to members of the CHIPPS study team, and external regulators if requested. The servers are protected by firewalls and maintained according to best practice. The physical location of the servers is protected by CCTV and security door access.

The database and associated code lists have been developed by the Study Coordinators in conjunction with NCTU. The database software (REDCap) provides a number of features to help maintain data quality, including: maintaining an audit trail, allowing custom validations on all data, allowing users to raise data-query requests, and search facilities to identify validation failure/missing data.

Once data entry is complete the database will be locked prior to any trial analysis or unblinding. The Data Management Team will provide a read-only link for the Trial Statistician to access the data. After completion of the study the database will be retained on the servers of NCTU for on-going analysis, for 10 years.

The screening and enrolment logs will remain at the care home. For recruitment monitoring purposes, identifiable patient information will be redacted, and pseudoanonymised copies of these logs being taken to the research office. Following consent, identifiable (consented participants only) screening data, linked to the Participant Identification Number, will be held locally at the University research office, in a locked filing cabinet. After completion of the study the identification, screening and enrolment logs will be securely archived at each University research office for 10 years, unless otherwise advised by NCTU.

### Sample size

A sample size of 880 (440 in each arm) would detect a decrease in fall rate from 1.50 per individual over 6 months to 1.178 with 80% statistical power. These assumptions are based upon data from the CAREMED [29] study, which found a fall rate of 1.5 per individual over a 6-month period and an intraclass correlation coefficient (ICC) no greater than 0.07 for the endpoint of interest. The detectable difference (from 1.5 to 1.178) is a relative reduction of 21% which is half that detected within a UK-based, pharmacist-led medication review service provided to care homes [30]. The CAREMED trial indicated a mortality rate of 33% and further loss to follow-up of 5% over 12 months. Thus, a reasonable estimate of total losses due to mortality or other reasons over 6 months would be 20%, and is taken into account in the above. However, we will use data, where possible, up to the point at which someone withdraws from the study.

To recruit 880 resident participants there will be a recruitment target of 44 triads, with a mean of 20 participants from each, a loss rate of no more than 20% and an ≤ ICC of 0.05.

### Statistical methods

An intention-to-treat analysis will be conducted. The primary outcome (‘falls per resident’) will mostly likely follow a Poisson distribution and a between-group comparison to estimate the difference in falls will be made using a Poisson Regression model. This model will include baseline fall rate, prognostic variables (specified prior to analysis) and group as a fixed factor. The unit of analysis will be the individual participant but, due to the study design incorporating ‘clustering’ these unit outcomes are likely to be correlated. Therefore, a Generalised Estimation Equation (GEE) approach will be used. The Poisson assumption will be assessed with ‘fit’ statistics and, if appropriate, a Zero Inflated Poisson, or a Poisson model with an over-dispersion term will be considered. An analogue GEE model will be used for secondary outcomes, with an appropriate change to the error distribution (e.g. Normal). The estimate of the between-group difference will be provided with a 95% confidence interval and tested at the 5% significance level.

There are currently no plans for any subgroup analyses.

### Safety reporting of Serious Adverse Events

The processes for the recording of SUSARs (Sudden Unexpected Serious Adverse Events), SAEs (Serious Adverse Events) and AEs (Adverse Events) and near misses in PIP documentation, GP and care home records, notification to NCTU, CI review, expedited and periodic reporting to REC will be documented in the study-specific Safety Management Plan.

For the purposes of this trial, SAEs are defined as inpatient hospitalisation and death. The expedited, i.e. immediate reporting is required if they are:

- *related* to the study (i.e. they resulted from the intervention) and
- *unexpected* (referred to hereafter as SUSARs)

A mixture of prospective and retrospective SUSAR notification will be used.

Prospective: from the beginning of the intervention until 30 days after the intervention ends GPs will be asked to report SUSARs immediately via a SUSAR Form to a dedicated NCTU safety email address.

Retrospective: a systematic retrospective collection of SAEs will be conducted in both intervention and control practices, whereby the NCTU Trial Manager will contact every participating care home once a month and ask about any SAEs. Deaths and hospitalisations in both arms will also be reported to the REC via the annual report.

The causality assessment of the SAE should be given by the GP. If the GP identifies a positive causality (i.e. the SAE is linked to the PIP intervention and is, therefore, a SUSAR) then this is signed off by the CI. The GP must assess the causality of all SAEs in relation to the PIP intervention using the definitions in the table below. If the event is classified as ‘serious’ and assessed as being related to the PIP intervention then a SUSAR Form must be completed and NCTU notified within 24 h.

All staff involved in the care of study participants (i.e. PIPs, care home staff, any other healthcare professionals) will also be asked to report, immediately, to a separate dedicated email address (chipps.safety@uea.ac.uk), any events about which they are concerned. NCTU can be notified of any further safety concerns or near misses by all staff involved in the care of study participants via a study-specific safety email address Table 2.

**Table 2:** Serious Adverse Event (SAE) causality definitions

Event type	Causality assessment	Description
SAE	Unrelated	There is no evidence or rationale for any causal relationship
SUSAR (Sudden Unexpected Serious Adverse Event)	Likely to be related	There is evidence, and a rationale, to suggest a causal relationship and other possible contributing factors can be ruled out

### Trial management

The trial is overseen by a Trial Management Group (TMG) comprising the Programme Chief Investigator, The Trial Co-Chief Investigators, the local Principal Investigators, the Senior Programme Manager, the NCTU Manager and the Programme Administrator. The trial is advised by a Programme Steering Committee (PSC) which provides expert oversight of the trial, making decisions as to the future continuation (or otherwise) of the trial, by monitoring recruitment rates, approving proposals by the TMG concerning any change to the design of the trial, as well as receiving letters of feedback from the independent Data Monitoring Committee (DMC). The DMC comprises a statistician, an academic pharmacist with an interest in patient safety, and an academic GP (Chair) with extensive trials’ experience. The DMC has a remit to monitor the safety of the trial participants through examination of trial safety and efficacy data, thereby providing advice to the Chair of the Programme Steering Committee (PSC). The DMC Chair informs the Chair of the PSC if, in the view of the DMC, one trial arm is clearly indicated or contraindicated (for all participants or a particular category of participants), and there is a reasonable expectation that this new evidence would materially influence patient management.

There is a study Quality Management and Monitoring Plan (version 2: 1 June.2018) which details the procedures for quality control and data monitoring by the NCTU. The study will also be subject to random monitoring by the host Universities and local Research and Development Departments.

## Discussion

The internal pilot study confirmed the feasibility of all study processes and no safety concerns were identified. The results will be reported in full when the main trial findings are published. Resident recruitment is ongoing and on target. The trial is expected to complete in early 2020. The TMG is grateful for the support of the TSC and DMC who have confirmed that to date there are no concerns.

The study should provide important information on the clinical and cost-effectiveness of involving pharmacists in general practices and care homes –a policy being widely rolled out across all home nations but with no RCT of evidence. The trial is part of a programme of work, part of which has been to develop a training programme for pharmacists involved in care homes and this will be made publicly available. The study also includes a detailed process evaluation and, taken together with the trial results, the findings should allow recommendations to be made about the optimum way to roll out and manage this wider role for pharmacists. At a time when the UK population is ageing, the results will be relevant both to care home residents and frail, community-dwelling older adults (Additional file 4).

## Supplementary information

**Additional file 1.** Service specification.

**Additional file 2.** Study recruitment documentation.

**Additional file 3.** Capacity assessment for residents.

**Additional file 4.** SPIRIT 2013 Checklist: Recommended items to address in a clinical trial protocol and related documents.

## Data Availability

Requests for access to the final trial dataset will be considered, and approved in writing where appropriate, after formal application to the TMG/PSC. Considerations for approving access are documented in the PMG/PSC Terms of Reference.

## Footnotes

1 Based on earlier work, we anticipate a consent rate of 55% and so would expect to invite approximately 35 residents/GP practice

2 The study will recruit residents both with and without capacity because this is representative of care home populations, where many residents are without capacity, and to exclude such residents would render the research ungeneralisable. In addition, all residents could benefit from the intervention, and so residents without capacity should not be denied the opportunity to participate
