# Supplementary material for: Protocol for a cluster randomised controlled trial to determine the effectiveness and cost-effectiveness of independent pharmacist prescribing in care homes: the CHIPPS study
Source: Trials. 2020 Jan 21;21:103. doi: 10.1186/s13063-019-3827-0 (PMC6975047; doi:10.1186/s13063-019-3827-0)
Supplement: Supplementary file 1 — Additional file 1. Service specification. [file 13063_2019_3827_MOESM1_ESM.docx]

## Appendix 1. Service Specification

**
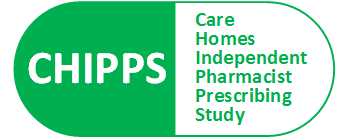
**

**Care Homes Independent Pharmacist Prescribing Study (CHIPPS)**

**Service Specification**

1. **Service outline**

CHIPPS is a National Institute for Health Research (NIHR) programme grant to develop and deliver a cluster randomised controlled trial to determine the effectiveness and cost-effectiveness of making pharmacist prescribers part of a team working alongside care home staff and General Practitioners (GPs) in care homes for older people. CHIPPS will provide a Pharmacist Independent Prescriber (PIP) to review and optimise prescribing in recruited residents and facilitate and support cost effective evidence based prescribing and medicines management in care homes for older people.

1. **Aims and objectives**

The aim of the service is to improve health outcomes and wellbeing of care home residents and ensure medicines are prescribed and managed in a safe, effective and cost effective way.

In order to meet the stated aims recruited GP practices and care homes will work with a PIP who has demonstrated competency in care home medicines management and prescribing in older people. The PIP will be based at the GP practice, for the duration of the study, and will have developed an excellent working relationship with the GP practice and care home prior to commencing the service delivery. The service will run for a period of 3 months.

**3. Inclusion/exclusion criteria for service**

**3.1 Pharmacist Independent Prescriber (PIP)**

Inclusion criteria

- Registered as a pharmacist independent prescriber
- Following training can demonstrate competence to deliver service (See section 4)
- Ability to work flexibly and commit a minimum of 16 hours a month to deliver the service for three months

Exclusion criteria

- Substantive employment with the community pharmacy (branch/store) which supplies medicines to the care home with which the PIP would work

**3.2 Care home**

Inclusion criteria

- Care Quality Commission (CQC) registered specialism as caring for adults over 65
- Primarily caring for residents over 65 years

Exclusion criteria

- Care homes who receive additional medication focussed services with a visit frequency ≥ monthly
- Care homes which only provide carer or support remotely (They do not have carers on site 24 hours a day)
- Care homes which are currently under formal investigation with the Care Quality Commission (CQC) or equivalent body

**3.3 Residents**

Inclusion criteria

- Resident under the care of the participating GP practice
- Residents currently prescribed at least 1 medicine
- Residents or their appropriate representative who are/is able to provide informed consent/assent
- Permanent resident in care home (not registered for respite care/temporary resident)
- Residents must be 65 years or over

Exclusion criteria

- Residents who are currently receiving end of life care (equivalent to yellow (stage C) of the Gold Standards Framework prognostic indicator)
- Resident with additional limitations on their residence (e.g. held securely)
- Participating in another research study

**4. Service requirements**

**4.1. Recruitment and employment of the Pharmacist Independent Prescriber (PIP)**

- Initial identification and recruitment of the PIP will be conducted by the CHIPPS management committee
  - The PIP will require:
    - Excellent interpersonal, communication and IT skills
    - Familiarity with relevant GP software systems
    - Experience of providing prescribing and medicines management advice and support
    - Previous experience of working in GP practice environment
    - Be able to travel to site locations
    - A mobile phone to be contactable for the purposes of delivering this service
    - Appropriate indemnity insurance for prescribing
- PIP will be employed according to local arrangements and seconded to the relevant GP practice for the duration of the study and during training and competency assessment (see section 4.2)

**4.2 Training and competency assessment of PIP**

See Appendix 1

**4.3 PIP roles and responsibilities [NB: categorised as essential or not]**

The PIP will, where appropriate:

4.3.1 Review each resident’s medication and develop and implement a pharmaceutical care plan^^[[1]](#footnote-1)^^ (essential)

- Optimise prescribing ensuring clear indication and evidence base for each medication (taking into consideration national and local pathways, guidelines and formularies), informed by tools such as STOPP/START
- Minimise the potential for adverse effects
- Optimise the dose of all medication
- Co-ordinate appropriate monitoring and associated tests for all medicines and conditions
- Agree initial care plan with GP, care staff and resident (where appropriate)
- Document and maintain records relating to review and care plan in GP and care home records as appropriate

4.3.2 Prescribing (essential)

- Authorise repeat prescriptions
- Co-ordinate appropriate monitoring and associated tests for all medicines and conditions
- Deprescribe medicines according to agreed pharmaceutical care plan
- Document medication changes in GP and care home records and notify supplying pharmacy of all changes to medication within 24 hours
- Only initiate new medicines for existing diagnoses or for common ailments which can be managed with medicines classified by the Medicines and Healthcare products Regulatory Agency (MHRA) as Pharmacy (P) or General Sales List (GSL)
- Any additional areas of prescribing must be agreed and documented with the GP practice prior to prescribing (e.g. antibiotics for simple UTIs)

4.3.3 Communication (essential)

- Agree local protocols for communication with GP practice and care home prior to commencing service. This should include:
  - Process of communication and messaging
  - The location and expected level of detail of all PIP interventions in the medical records Process and communication of referrals for activities outside the competence of the PIP
- Inform supplying community pharmacy about service and role (prior start of service)
  - Communicate all changes in medication to supplying pharmacy
- Complete all documentation and recording of activities as required by the study team

4.3.4 Support systematic ordering, prescribing, and administration processes with each care home, GP practice and supplying pharmacy where needed: (undertaken at PIP’s discretion)

- Provide instructions on how to administer each drug
- Synchronise residents prescription quantities for monthly cycles
- Add or clarify directions for all medication where it is currently not clear
- Provide advice on repeat prescription ordering processes to:
  - Minimising missed items
  - Optimising quantities
- Optimise the use of homely remedies within the care home
- Reconcile resident medication following a transfer of care

4.3.5 Training provision (undertaken at PIP’s discretion)

- Review training needs of care home and GP practice and draft proposed training plan
- Provide training to care home staff on training needs basis from agreed list of potential topics/areas
- Provide guidance to relevant GP practice on training needs basis from agreed list of potential topics/areas

**4.4 Safe and effective service provision**

- PIP will be contactable and respond to messages within 24 hours (Monday - Friday)
- The PIP will establish a locally agreed protocol with the GP practice for referral/notification of all medicine related queries from CHIPPS participants to the PIP as appropriate (see 4.3.5)
- PIP will have full (read/write) access to GP record system to issues prescriptions and update records
- Where possible PIP will use remote access to update records when changes are made to GP held record
  - Where remote access is not feasible the PIP must update records within 24 hours of making a change
- PIP will have full (read/write) access to care home records to update records during all visits using appropriate local reporting systems
- The PIP will visit/contact the care home at least once a week
- The PIP will visit/contact the GP practice at least once a week
- Wherever possible, all annual leave should be agreed before the beginning of the study. A clear system for transfer of responsibility communicated to GP, care home and supplying pharmacy
- The PIP will work within the local prescribing formularies of GP practice and primary care organisation.
- The PIP will report and document all significant clinical events or near misses using local reporting procedures and study documentation.
- Ensure all records are aligned

**5. Outcomes from service**

As part of the feasibility study, we will measure levels of resource-use associated with the PIP intervention, which will be estimated using the PIP log (Appendix 2), which the PIP will complete every day.

**6. End of service transitional arrangements**

The duration of service will be clearly documented in study documentation and signed agreement to service provision completed by Care Home and GP practice prior to commencing the study.

- All original policy and procedure documentation will be kept prior to amendments made during service provision
- Transfer meeting with PIP, GP practice and care home at least 3 weeks prior to end of service
  - Agree transfer of responsibilities from PIP
    - Agree named contact point for medication issues at GP practice
  - Communicate current plans for each resident
    - Transfer of care plan and set review date
  - Agree changes in policy and procedures

1. A Pharmaceutical care plan is defined as plan for the responsible provision of medicine-related care for the purpose of achieving defined outcomes that improve the patient’s quality of life. It involves gathering information, identifying problems, assessing problems and desired improvements. [↑](#footnote-ref-1)
