# Supplementary material for: Protocol for a cluster randomised controlled trial to determine the effectiveness and cost-effectiveness of independent pharmacist prescribing in care homes: the CHIPPS study
Source: Trials. 2020 Jan 21;21:103. doi: 10.1186/s13063-019-3827-0 (PMC6975047; doi:10.1186/s13063-019-3827-0)
Supplement: Supplementary file 2 — Additional file 2. Study recruitment documentation. [file 13063_2019_3827_MOESM2_ESM.docx]

Appendix 2 Study Recruitment documentation

Draft letter of invitation for PIPs (send by email from the network)

(Version 1, 08.08.2017)

EMAIL SUBJECT:

CHIPPS Study - pharmacists sharing responsibilities in care homes

We would like to invite you to participate in a randomised controlled trial.

A team of health care professionals, patient representatives and researchers (funded by an NIHR Programme Grant) have developed a new service involving specially trained prescribing pharmacists taking on more responsibility for medicines in care homes.

If you were to participate, you would be paired with a GP practice, and randomised to one of two groups:

The intervention group – you would complete an intensive (two day) training programme in the CHIPPS service specification and then assume responsibility, in collaboration with the GP, for the medicines management of up to 20 care home residents, for six months.

The control group – you would not deliver the intervention, but the training will be available on-line and by Webinar, after the intervention period.

If you are interested then please read the attached participant information sheet and consent form and get back to us; we’ll then be able to answer your questions.

Many thanks.

Professor David Wright & Professor Richard Holland

UEA


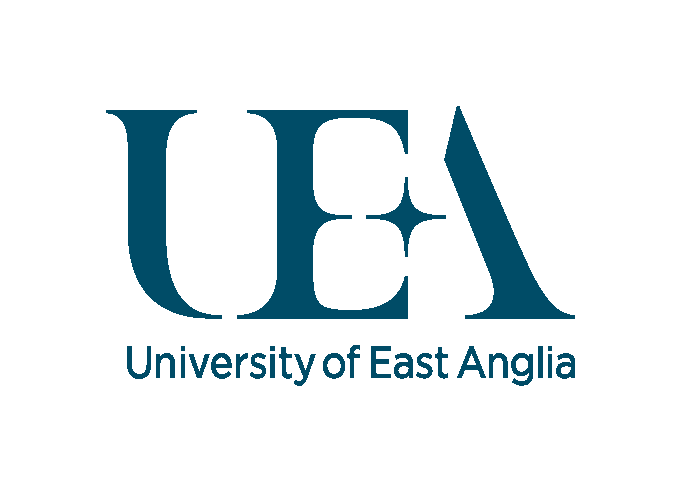


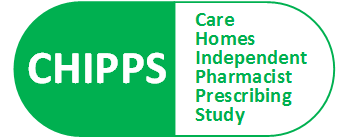

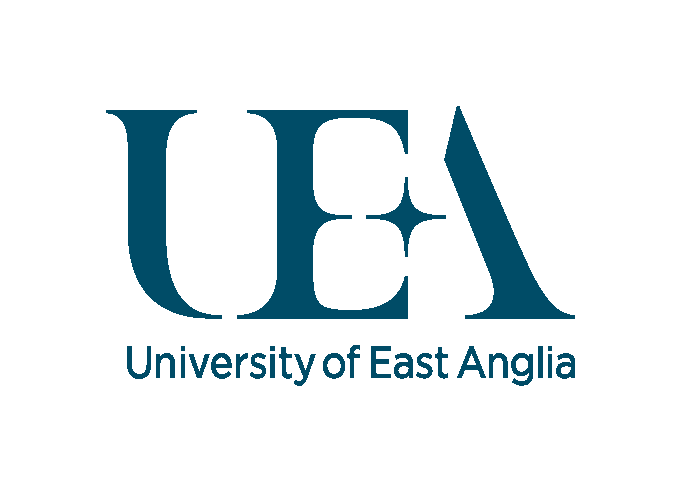


# CHIPPS Work Package 6

# PARTICIPANT INFORMATION SHEET: PIP

We would like to invite you to take part in this research study. The study is being run by the University of East Anglia, together with the University of Aberdeen, Queen's University of Belfast and the University of Leeds. The study is funded by the UK National Institute for Health Research. Before you decide we would like you to understand why the research is being done and what it would involve for you.

**What is the purpose of the study?**

We have developed a new service in which a specially trained Pharmacist Independent Prescriber (PIP) will become part of the care home team, working alongside general practitioners, to improve the use of medicines which we hope may improve health and wellbeing of residents and ensure medicines are prescribed and managed in a safe, effective and cost effective way. In this study we wish to test how effective the service that we have developed is. It has been tested in a small feasibility study which helped us refine the details of the service, which we will now evaluate in this large multi-centre randomised controlled trial.

**Why have I been invited?**

We are inviting prescribing pharmacists to participate in the study in the delivery of the intervention.

**Do I have to take part?**

You are not obliged to participate. It is up to you to decide to join the study, or not. If you agree to take part, you are free to withdraw at any time, without giving a reason.

**What will happen to me if I take part?**

If you take part, you would then be paired with a GP and care home(s). You, and your GP and care home(s), would be put at random, into one of two groups – one delivering the intervention and the other not, as a control.

If you are in the intervention group, you will complete an intensive two day training programme in the CHIPPS service specification (and research procedures and study documents) and following successful completion of this, and with evidence of competency, you would begin delivering the intervention, three months after the training.

To fully prepare you for this role, you would also have time within the GP practice and associated care home (two days in total) familiarising yourself with systems and usual practice. You would assume responsibility of up to 20 care home residents for the six months of the study, in accordance with the CHIPPS service specification; you would have half a day a week available for this. You would agree with the GP how to report back your activities in the care home. All the time spent on this work will be funded by the study.

If you are assigned to the control group, you would not deliver the intervention, but the training will be available to you at the end of the intervention period on-line and also with Webinars.

**What are the possible benefits of taking part in this study?**

You would have the time to optimise the participating patients’ medicines, which should be beneficial to the patients and also to the GP practice, in the form of GP time saved. This would also be a valuable contribution to your personal and professional development.

**What disadvantages are there?**

We do not think there are any disadvantages for you in taking part.

**What happens when the study comes to an end?**

The PIP will prepare the care home(s) and practice staff for their departure by making provision for a thorough and effective handover.

These findings will inform future NHS recommendations for the role of pharmacists. We aim to publish the results of this project both locally and nationally. These reports will not include names or other personal details that would allow you to be identified.

**What if there’s a problem?**

If you have any concerns about the study, you should first raise them directly with the researchers (details below). If you have further concerns please contact the Sponsor’s representative Clare Symms, Research Governance Manager, Norfolk & Suffolk Primary & Community Care Research Office, Hosted by South Norfolk CCG, Lakeside 400, Old Chapel Way, Broadland Business Park, Thorpe St Andrew, Norwich, NR7 0WG Direct Dial - 01603 257020

**Who has reviewed the study?**

The study has been approved by East of England, Cambridge Central NHS Research Ethics Committee.

**What will happen to the data collected?**

South Norfolk CCG is the sponsor for this study based in the United Kingdom. We will be using information from you and your medical records in order to undertake this study and will act as the data controller for this study. This means that we are responsible for looking after your information and using it properly.

Your rights to access, change or move your information are limited, as we need to manage your information in specific ways in order for the research to be reliable and accurate. If you withdraw from the study, we will keep the information about you that we have already obtained. To safeguard your rights, we will use the minimum personally-identifiable information possible.

You can find out more about how we use your information at: <http://www.southnorfolkccg.nhs.uk/public-information/fair-processing-notice>

The local participating organisation will collect information from you and your medical records for this research study in accordance with our instructions.

The local participating organisation will keep your name and contact details confidential and will not pass this information to the sponsor, South Norfolk CCG. The local participating organisation will use this information as needed, to contact you about the research study, and make sure that relevant information about the study is recorded for your care, and to oversee the quality of the study.

The research team will keep identifiable information about you from this study for 10 years after the study has finished until 30/04/2030.

South Norfolk CCG will collect information about you for this research study from the local participating organisation. The local participating organisation will not provide any identifying information about you to South Norfolk CCG. We will use this information to enable research, which is in the public interest. Certain individuals from South Norfolk CCG, Norwich CTU and regulatory organisations may look at your medical and research records to check the accuracy of the research study. The people who analyse the information will not be able to identify you and will not be able to find out your name or contact details.

**Further information and contact details**

If you would like further information, please contact one of the researchers:

Annie Blyth/Viv Maskrey, Programme Co-ordinators,

University of East Anglia, Norwich. NR4 7TJ.

Tel: 01603 593308 / 593966, Email: [a.blyth@uea.ac.uk](mailto:a.blyth@uea.ac.uk) or [v.maskrey@uea.ac.uk](mailto:v.maskrey@uea.ac.uk)

**PIP - CONSENT FORM**

(Version 3, 01/07/2018)

### Title of Study: CHIPPS WP6

### CHIPPS Care Homes Independent Pharmacist Prescribing Study

**REC ref: 17-0360**

**Name of Researcher**:

**Name of PIP Participant:**

**Participant Identification Number:**

**Please initial box**

1. I confirm that I have read and understand the information sheet version number 2, dated 01/07/2018 for the above study and have had the opportunity to ask questions.

2. I understand that my participation is voluntary and that I am free to withdraw at any time, without giving any reason, and without my legal rights being affected. I understand that should I withdraw then the information collected so far cannot be erased and that this information may still be used in the project analysis.

3. If randomised to the Intervention Group I agree to receive CHIPPS training and following this training to deliver the CHIPPS package as described in the CHIPPS Service Specification

4. I agree to facilitate CHIPPS research processes in the care home and GP Practice

5. I agree to my contact details and a copy of this consent form being held securely and confidentially by CHIPPS coordinating centre/Norwich Clinical Trials Unit.

6. I am happy to be contacted about participating in additional research interviews which are part of this study

7. I agree to take part in the above study.

**_____________________ ______________ ________________________­­­_­­­_**

# Name of PIP Participant Date Signature

**_______________________ ______________ __________________________**

Name of Person taking consent Date Signature

4 copies:

1 for participant, 1 for location file (original), 1 for GP practice and 1 for Norwich CTU Trial Office

Expression of interest letter to GP practice

CHIPPS Study - pharmacists sharing responsibilities for residents’ medicines in care homes

We would like to invite all practices who provide care to patients in care homes (ideally 35 or more) to express an interest to participate in a study. If you do not provide care for patients in care homes (residential or nursing), no further action is required on your part.

A team of health care professionals, patient representatives and researchers (funded by an NIHR Programme Grant) have developed a research study to explore the impact of involving pharmacists taking on more responsibility for medicines in care homes.

In conjunction with general practices (GPs) and other stakeholders, we have developed a new service in which a specially trained ‘pharmacist independent prescriber’ (PIP) will become part of the care home team, working alongside GPs, to improve the use of medicines. We hope this will improve residents’ health outcomes and wellbeing, as residents will be closely monitored and reviewed.

We have already tested this new service in a feasibility study which has helped us refine the details of the service, and we are now evaluating the effectiveness and cost-effectiveness of the service in a large multi-centre randomised controlled trial (RCT), with an internal pilot study. The pilot is currently underway, and we are now inviting practices to express an interest in participating in the main RCT. GP practices who agree to participate in the study will subsequently be randomly selected to either deliver the CHIPPS service to participating care home residents (intervention group), or not deliver the service (control group).

If your practice were to participate, we would ask you to identify eligible care home residents (aged 65 or over and prescribed at least one medicine) in one or two care homes for which you provide care and facilitate the research team in liaising with the care home and collecting data.

You would be asked to work with a prescribing pharmacist, on a sessional basis, based in your practice (who will have received study specific training) to provide a new medicine management service to these patients for six months. If you do not already have a practice based pharmacist with prescribing qualifications the study will provide you with one. If you do have a prescribing pharmacist, participation in the study could offer them the opportunity of completing the CHIPPS training package.

Practices will receive service support costs from the (Local Clinical Research Network) CRN to help identify patients plus a standard fee for other research activities. I have attached a RISP with more details.

If you are interested in participating in this study then please complete and return the attached Expression of Interest form.

We will then contact you to provide further information and to answer any questions you may have.

Many thanks

Professor David Wright, University of East Anglia


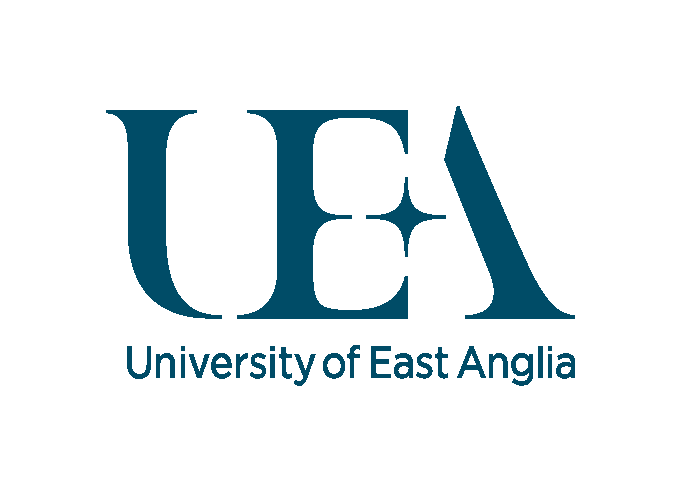


# PARTICIPANT INFORMATION SHEET: GP PRACTICE

We would like to invite you to take part in this research study. The study is being run by the University of East Anglia, together with the University of Aberdeen, Queen's University of Belfast and the University of Leeds. The study is funded by the UK National Institute for Health Research. Before you decide we would like you to understand why the research is being done and what it would involve for you.

**What is the purpose of the study?**

We have developed a new service in which a specially trained Pharmacist Independent Prescriber (PIP) will become part of the care home team, working alongside general practitioners, to improve the use of medicines which we hope may improve health outcomes and wellbeing of residents and ensure medicines are prescribed and managed in a safe, effective and cost-effective way. We have tested the service in a small feasibility study which has helped us refine the details of the service which we will now evaluate in this large multi-centre randomised controlled trial; this is what we are asking you to participate in.

**Why have I been invited?**

We are inviting GP practices that are responsible for sufficient residents to recruit 20 participants ideally in 1–2 care homes, who are registered as patients of your practice.

**Do I have to take part?**

You are not obliged to participate. It is up to you to decide to join the study, or not. If you agree to take part, you are free to withdraw at any time, without giving a reason.

**What will happen to me if I take part?**

If you take part, you would then be paired with a PIP. We would ask you to identify suitable care home(s) and we would contact the manager of your care home(s), on your behalf, with information about the study, and a letter inviting the care home to participate. If the care home does not agree, we would contact another one of your care homes.

To recruit patients, we would ask you to write a letter to all your eligible patients (>65 years and on at least one medicine) resident in the home(s), telling them about the study and asking if they’d like to participate (we would provide the details for the letter and information). We would ask the care home manager to deliver the letters and ask the residents if a study researcher could talk with them. If they agree to this, a study researcher would visit them and, if the resident is willing, undertake informed consent. If a patient does not have the capacity to consent, we would contact an appropriate third party, such as a relative, to act as Consultee.

At this point you (your paired PIP and your care home/s) would be put at random into one of two groups – one delivering the intervention and the other not, as a control. If you are in the intervention group, your PIP would receive two days’ intensive training to prepare them for the role; the intervention would begin three months after the training. In addition, to enable you to be confident that the PIP would work in alliance with you, and your wishes and practice policies, they would spend some time with the care home and also with you and your practice (two days in total) familiarising themselves with systems and normal practice. Then they would be equipped to assume the responsibility of your care home residents for the six months of the trial - they will have half a day a week available for this. You would agree with the PIP, how you would want them to report back their activities to you. The cost of the PIP’s time spent on this work will be covered by the NHS and research costs. In the intervention group the PIP would assume responsibility for managing the participating patients’ medicines, according to the CHIPPS service specification which utilises a Pharmaceutical Care Plan (PCP).

In the control group care would continue as usual, we would ask you to facilitate a researcher to extract routine data on prescribing and health service resource use from primary care records at baseline and at six months (this will also be a requirement for the intervention group patients).

**What are the possible benefits of taking part in this study?**

The PIP would have the time to optimise your participating patients’ medicines, which should be beneficial to the patients and also to your practice, in the form of GP time saved.

**What disadvantages are there?**

We do not think there are any disadvantages of taking part beyond the time taken to initiate participation and the on-going communication with the PIP.

**What happens when the study comes to an end?**

The PIP will prepare the care home and practice staff for their departure by making provision for a thorough and effective handover.

These findings will inform future NHS recommendations for the role of pharmacists. We aim to publish the results of this project both locally and nationally. These reports will not include names or other personal details that would allow you to be identified.

**What if there’s a problem?**

If you have any concerns about the study, you should first raise them directly with the researchers (details below). If you have further concerns please contact the Sponsor’s representative Clare Symms, Research Governance Manager, Norfolk & Suffolk Primary & Community Care Research Office, Hosted by South Norfolk CCG, Lakeside 400, Old Chapel Way, Broadland Business Park, Thorpe St Andrew, Norwich, NR7 0WG, direct dial - 01603 257020.

**Who has reviewed the study?**

The study has been approved by East of England, Cambridge Central NHS Research Ethics Committee.

**What will happen to the data collected?**

South Norfolk CCG is the sponsor for this study based in the United Kingdom. We will be using information from you and your medical records in order to undertake this study and will act as the data controller for this study. This means that we are responsible for looking after your information and using it properly.

Your rights to access, change or move your information are limited, as we need to manage your information in specific ways in order for the research to be reliable and accurate. If you withdraw from the study, we will keep the information about you that we have already obtained. To safeguard your rights, we will use the minimum personally-identifiable information possible.

You can find out more about how we use your information at: <http://www.southnorfolkccg.nhs.uk/public-information/fair-processing-notice>

The local participating organisation will collect information from you and your medical records for this research study in accordance with our instructions.

The local participating organisation will keep your name and contact details confidential and will not pass this information to the sponsor, South Norfolk CCG. The local participating organisation will use this information as needed, to contact you about the research study, and make sure that relevant information about the study is recorded for your care, and to oversee the quality of the study.

The research team will keep identifiable information about you from this study for 10 years after the study has finished until 30/04/2030.

South Norfolk CCG will collect information about you for this research study from the local participating organisation. The local participating organisation will not provide any identifying information about you to South Norfolk CCG. We will use this information to enable research, which is in the public interest. Certain individuals from South Norfolk CCG, Norwich CTU and regulatory organisations may look at your medical and research records to check the accuracy of the research study. The people who analyse the information will not be able to identify you and will not be able to find out your name or contact details.

**Further information and contact details**

If you would like further information, please contact one of the researchers:

Annie Blyth/Viv Maskrey, Programme Co-ordinators, UEA, Norwich, NR4 7TJ.

Tel: 01603 593308 / 593966, Email: [a.blyth@uea.ac.uk](mailto:a.blyth@uea.ac.uk) or [v.maskrey@uea.ac.uk](mailto:v.maskrey@uea.ac.uk)

**GP - CONSENT FORM**

(Version 1, 8^th^ August 2017)

### Title of Study: CHIPPS WP6 Study

### Care Homes Independent Pharmacist Prescribing Study

**REC ref: XX/XX/XXXX**

**Name of Researcher**:

**Name of GP:**

**Name of GP Practice:**

**Participant Identification Number:**

**Please initial box**

1. I confirm that I have read and understand the information sheet version number X, dated xx/xx//2017 for the above study and have had the opportunity to ask questions.

2. I understand that my participation is voluntary and that I am free to withdraw at any time, without giving any reason, and without my medical care or legal rights being affected. I understand that should I withdraw then the information collected so far cannot be erased and that this information may still be used in the project analysis.

3. I agree to facilitate CHIPPS research processes in the practice by contacting the care home(s) where some of the practice’s patients are resident

4. I agree to facilitate CHIPPS research processes in the practice by supporting and mentoring the assigned CHIPPS Independent Prescribing Pharmacist (PIP)

5. I agree to facilitate any other CHIPPS research processes, such as data collection by researchers/PIP

6. I agree to my contact details and a copy of this consent form being held securely and confidentially by CHIPPS coordinating centre/Norwich Clinical Trials Unit.

7. I am happy to be contacted about participating in additional research interviews which are part of this study

8. I agree to take part in the above study.

**_____________________ ______________ ________________________­­­_­­­_**

# Name of Participant GP Date Signature

**_______________________ ______________ __________________________**

Name of Person taking consent Date Signature

4 copies:

1 for GP signatory, 1 for location file (original), 1 for GP Practice study file and 1 for Norwich CTU Trial Office

Letter of invitation for Care Home Managers (sent by GP Practice)

(Version 1, 8^th^ August 2017)

EMAIL SUBJECT:

CHIPPS Study - pharmacists sharing responsibilities in care homes

We would like to invite your care home to participate in a study.

A team of health care professionals, patient representatives and researchers (funded by an NIHR Programme Grant) have developed a new service involving specially trained prescribing pharmacists taking on more responsibility for medicines in care homes. We are working with this research team to test the intervention, and would like to invite your care home to participate.

If you were to participate, we would be paired with a specially trained prescribing pharmacist (PIP). We would then be randomised into one of two groups:

The intervention group – the PIP would complete an intensive (two day) training programme in the CHIPPS service specification and then assume responsibility, in collaboration with myself, for the medicines management of up to 20 care home residents, for six months.

The control group – we would not deliver the intervention, and your residents would receive their normal care from me.

I hope you are interested in working on this with us, so please read the attached information sheet and consent form and complete and get back to us.

Then we will get back to you and answer any questions.

Many thanks.

Dr XXXXXXX

XXXXX Medical Practice


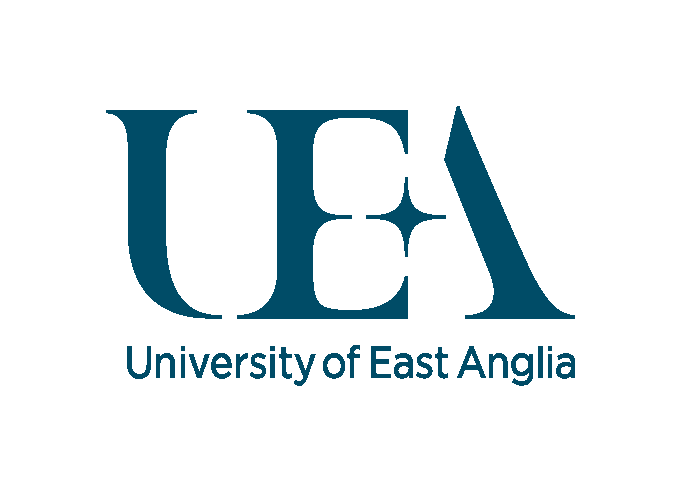


**PARTICIPANT INFORMATION SHEET: CARE HOME**

We would like to invite you to take part in this research study. The study is being run by the University of East Anglia, together with the University of Aberdeen, Queen's University of Belfast and the University of Leeds. The study is funded by the UK National Institute for Health Research. Before you decide we would like you to understand why the research is being done and what it would involve for you.

**What is the purpose of the study?**

We have developed a new service in which a specially trained Pharmacist Independent Prescriber (PIP) will become part of the care home team, working alongside general practitioners, to improve the use of medicines which we hope may improve health outcomes and wellbeing of the care of residents and ensure medicines are prescribed and managed in a safe, effective and cost-effective way.

In this study we wish to test how effective the service that we have developed is in practice. XXXX medical practice, which has patients resident in your home, is participating in this project and so we would like to invite your care home to facilitate the project in your care home.

**Why have I been invited?**

We are inviting care homes where a participating GP practice is responsible for patients who are resident in your care home.

**Do I have to take part?**

You are not obliged to participate. It is up to you to decide to join the study, or not. If you agree to take part, you are free to withdraw at any time, without giving a reason.

**What will happen to me if I take part?**

If you take part, the XXXX medical practice would send a letter inviting their patients, who are resident in your care home, along with some information about the study. We would then ask you to ask these residents if they would be prepared to talk to one of the research team. If the resident agrees, then a member of the research team will visit the home and talk to the residents and, if they are willing, they would be recruited onto the study. Of course, if a resident isn’t interested in participating that is absolutely fine; we would simply thank them for their time and they would not be approached again. If a resident does not have the capacity to consent, we would contact an appropriate third party, such as a relative, to act as Consultee.

Then, in collaboration with Dr XXXX, you would be paired with a specially trained PIP for the duration of the study and at this point you, along with your GP and PIP, would be put at random into one of two groups – one delivering the intervention and the other not, as a control.

If you are in the intervention group, your PIP would receive two days’ intensive training to prepare them for the role; the intervention would begin three months after the training. The PIP would also spend some time with the care home and also with Dr XXXX familiarising themselves with your home and systems. Then they would be equipped to assume, in collaboration with Dr XXXX, the responsibility of your care home residents for the six months of the study - they will have half a day a week available for this. You would agree with the PIP how you would want them to report back their activities to you. All the PIP’s time spent on this work would be paid for by the study.

If you are in the control group, the PIP would not come and deliver the intervention, but your residents would still be very valuable participants in the study.

Whatever group your home is in, the researcher would look at the participating residents’ records to record their medicines and the number of times they have seen their GP or any other health care providers. The researcher would also ask the care home staff to tell us how participants are doing using standard questionnaires.

**What are the possible benefits of taking part in this study?**

The PIP would have the time to optimise your participating patients’ medicines, which should be beneficial to the patients. In addition they would be available to assist in any medicines management issues that you might welcome help with, and would also be on call to answer acute issues.

**What disadvantages are there?**

We do not think there are any disadvantages of taking part beyond the time taken to initiate participation and the on-going communication with the PIP.

**What happens when the study comes to an end?**

The PIP would prepare the care home and practice staff for their departure by making provision for a thorough and effective handover.

These findings would inform future NHS recommendations for the role of pharmacists. We aim to publish the results of this project both locally and nationally. These reports will not include names or other personal details that would allow you to be identified.

**What if there’s a problem?**

If you have any concerns about the study, you should first raise them directly with the researchers (see details below). If you have further concerns please contact the Sponsor’s representative Clare Symms, Research Governance Manager, Norfolk & Suffolk Primary & Community Care Research Office, Hosted by South Norfolk CCG, Lakeside 400, Old Chapel Way, Broadland Business Park, Thorpe St Andrew, Norwich, NR7 0WG, Direct Dial - 01603 257020.

**What will happen to the data collected?**

South Norfolk CCG is the sponsor for this study based in the United Kingdom. We will be using information from you and your medical records in order to undertake this study and will act as the data controller for this study. This means that we are responsible for looking after your information and using it properly.

Your rights to access, change or move your information are limited, as we need to manage your information in specific ways in order for the research to be reliable and accurate. If you withdraw from the study, we will keep the information about you that we have already obtained. To safeguard your rights, we will use the minimum personally-identifiable information possible.

You can find out more about how we use your information at: <http://www.southnorfolkccg.nhs.uk/public-information/fair-processing-notice>

The local participating organisation will collect information from you and your medical records for this research study in accordance with our instructions.

The local participating organisation will keep your name and contact details confidential and will not pass this information to the sponsor, South Norfolk CCG. The local participating organisation will use this information as needed, to contact you about the research study, and make sure that relevant information about the study is recorded for your care, and to oversee the quality of the study.

The research team will keep identifiable information about you from this study for 10 years after the study has finished until 30/04/2030.

South Norfolk CCG will collect information about you for this research study from the local participating organisation. The local participating organisation will not provide any identifying information about you to South Norfolk CCG. We will use this information to enable research, which is in the public interest. Certain individuals from South Norfolk CCG, Norwich CTU and regulatory organisations may look at your medical and research records to check the accuracy of the research study. The people who analyse the information will not be able to identify you and will not be able to find out your name or contact details.

**Who has reviewed the study?**

The study has been approved by East of England, Cambridge Central NHS Research Ethics Committee.

**Further information and contact details**

If you would like further information, please contact one of the researchers:

Annie Blyth/Viv Maskrey, Programme Co-ordinators, UEA, Norwich. NR4 7TJ

Tel: 01603 593308 / 593966, Email: [a.blyth@uea.ac.uk](mailto:a.blyth@uea.ac.uk) or [v.maskrey@uea.ac.uk](mailto:v.maskrey@uea.ac.uk)

**CONSENT FORM – CARE HOME**

(Version 1, 08.08.2017)

### Title of Study: CHIPPS WP6

### Care Homes Independent Pharmacist Prescribing Study

**REC ref:**

**Name of Researcher**:

**Name of Care Home Manager:**

**Name of Care Home:**

**Care Home Identification Number:**

**Please initial box**

1. I confirm that I have read and understand the information sheet version number X, dated xx/xx/xx for the above study and have had the opportunity to ask questions.

2. I understand that my participation is voluntary and that I am free to withdraw at any time, without giving any reason, and without my legal rights being affected. I understand that should I withdraw then the information collected so far cannot be erased and that this information may still be used in the project analysis.

3. I agree to facilitate CHIPPS research processes in the care home

4. I agree to my contact details and a copy of this consent form being held securely and confidentially by CHIPPS coordinating centre/Norwich Clinical Trials Unit.

5. I am happy to be contacted about participating in additional research interviews which are part of this study

Yes

No

6. I agree to take part in the above study.

**_____________________ ______________ ________________________­­­_­­­_**

# Name of Care Home manager Date Signature

**_______________________ ______________ __________________________**

Name of Person taking consent Date Signature

4 copies:

1 for participant, 1 for location file (original), 1 for care home study file and 1 for Norwich CTU Trial Office


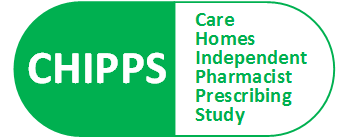


[XX PRACTICE – HEADED PAPER]

Letter from GP Practice to Care Home Resident

(Version 2 02/10/2017)

[Patient name & address]

[Date]

Dear [patient name],

The doctors at XX practice are participating in a research project called CHIPPS. The aim of the project is to study a service to help patients get the best possible benefit from their medicines. The service involves a specially qualified pharmacist with extra training, working within the practice, and sharing responsibility with the GP for prescribing medicines. The service is specifically for patients such as yourself who live in the XX home and take at least one medicine regularly. We have identified you as one of those patients and are writing to you to ask if you would like to help us with our research.

We have enclosed an information sheet so that you can read more. This explains that GP practices taking part will be divided into two groups. Only one of these groups will deliver the new service to their patients. The other group will continue to provide care as usual to their patients without the extra input from the pharmacist. Comparing what happens in each group allows us to assess the added value of the pharmacist’s input. At the moment we don’t know which group we will be in.

In the next few days [XX name] will be visiting you to find out if you would be interested in taking part. If you don’t want to speak to her/him please let the care home staff know.

You are free not to take part, and may withdraw at any time without stating a reason. This will not affect your medical treatment in any way.

If you would like any further information, please talk to [XX name] when they visit the care home or speak to [XX name] Care Home manager.

Yours sincerely,

Dr XX


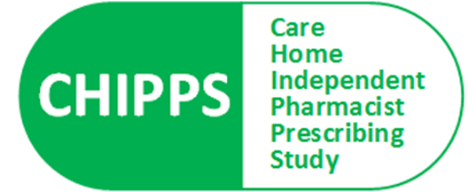

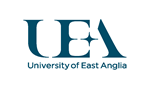


**We invite you to take part in our research study**

**CHIPPS**

**Care Homes Independent Pharmacist Prescribing Study**

**Can you help?**

# Resident’s Invitation to participate in a research study

This leaflet tells you about our study. This is a study which involves a qualified pharmacist working in partnership with your GP.

**Part 1: Essential information**

The pharmacist has had extra training to be able to prescribe medicines. They are called Pharmacist Independent prescribers (PIPs). Nationally PIPs are increasingly working within GP practices to help patients get the best from their medicines. During the study the pharmacist will share responsibility with your GP for prescribing your medicines.

The study is NOT testing any new pharmacist activity or experimental drugs; nor does it involve any extra blood tests or hospital visits, in addition to those that you may already receive. We want to be sure you understand the study before you decide to take part. Feel free to ask any questions.

**We are inviting people who live in this care home, and take at least 1 medicine regularly, to take part in our study.**

We want to see if a PIP, working in partnership with their GP, can help patients to get the best possible benefit from their medicines, and also keep an eye on their symptoms. In particular, we are interested to see whether this prevents people from having problems with their medicines and if it is a more effective way of managing medicines for people living in care homes.

If you agree to take part, you will be put at random into one of two groups (as if by ‘the toss of a coin’). The people who are put into group 1 will receive the new service; those in group 2 will not receive the new service, and their care will continue as normal. You will not be told which group you are in.

**Before you decide whether to take part, here are some answers to questions you may have:**

You do not have to take part and you may withdraw from the study at any time without stating a reason. This will not affect your legal rights, medical treatment or social care in any way; any data already collected for the study, will still be used

| **What happens if I am in the group that receives the new service?** |
| --- |
| During the next 6 months the PIP who is part of your GP practice’s staff, will take responsibility for your medicines.  The PIP may arrange to visit you to discuss how you are and to talk to you about your medicines and any problems you have with them. The PIP will explain a little about your medicines, and give brief advice if needed. The PIP will work with the staff at the care home to help you get the best from your medicines.  Occasionally, the PIP may suggest that changes are made to the medicines you are taking. As they are prescribers, the PIP will be able to organise this and will make a record of any changes on your medical records at the GP practice so that everyone involved in your care knows about the changes. If you are suddenly unwell you will continue to receive your normal service from your GP. |

| **What else happens if I take part?** |
| --- |
| Whichever group you are in, a researcher will visit you at the care home, and ask you to complete a very simple memory assessment, fill out a consent form to take part in the study and ask you some simple questions about your health. The researcher will come back and repeat some of these questions after 3 months and 6 months. The researcher’s visit will take about 30 minutes.  The researcher will also ask your permission to look at your records held in the care home and the GP practice, and write down your medical history, the medicines you have been prescribed and the number of times you have seen the GP or other health care providers. The researcher will also ask the Care Home staff to tell us how you are doing using standard questionnaires. |

| **What possible benefits are there to taking part?** |
| --- |
| PIPs that have been specially trained to work with GPs may be able to help people get the best from their medicines; this is something pharmacists are experienced in and are used to doing. |

| **What disadvantages are there?** |
| --- |
| Any advantages or disadvantages are unknown at present, which is why the service will be tested in this study. We do not think there are any disadvantages. However, you may not want to have the PIP prescribing your medicines, in which case you will probably not want to take part. |

| **What happens when the study comes to an end?** |
| --- |
| The intervention will not be provided at the end of the six month study period, when participants' medication will again become the sole responsibility of the GP.  However, individual areas may decide to continue the service, at their own discretion, or in accordance with national initiatives. These findings will also inform future NHS recommendations for the role of pharmacists. We aim to publish the findings of this study both locally and nationally. These reports will not include names or other personal details that would allow you to be identified. |

| **Confidentiality – Who will know information about me from this study?** |
| --- |
| All those involved will treat personal information in absolute confidence. Information about the medicines you are taking, the number of times that you see the PIP, GP and other health professional will be passed only to the research staff working on the study. |
| **Who is organising and funding the study?** |
| The study is funded by the NHS through the National Inst. of Health Research [RP-PG-0613-20007]. The study is being co-ordinated by researchers at the University of East Anglia, with research partners at the Universities of Aberdeen, Leeds and Queen’s University Belfast. |

| **What if there’s a problem?** |
| --- |
| If you have any concerns about the study, you should raise them first directly with the researcher (see details below).  If you have further concerns please contact the Sponsor’s representative Clare Symms, Research Governance Manager, Norfolk & Suffolk Primary & Community Care Research Office, Hosted by South Norfolk CCG, Lakeside 400, Old Chapel Way, Broadland Business Park, Thorpe St Andrew, Norwich, NR7 0WG 01603 257020 |

| **Who has assessed the ethical implications of the research?** |
| --- |
| The research has been approved by East of England NHS Research Ethics Committee. |

| **What will happen to the data collected?** |
| --- |
| South Norfolk CCG is the sponsor for this study based in the United Kingdom. We will be using information from you and your medical records in order to undertake this study and will act as the data controller for this study. This means that we are responsible for looking after your information and using it properly.  Your rights to access, change or move your information are limited, as we need to manage your information in specific ways in order for the research to be reliable and accurate. If you withdraw from the study, we will keep the information about you that we have already obtained. To safeguard your rights, we will use the minimum personally-identifiable information possible.  You can find out more about how we use your information at: <http://www.southnorfolkccg.nhs.uk/public-information/fair-processing-notice>  The local participating organisation will collect information from you and your medical records for this research study in accordance with our instructions.  The local participating organisation will keep your name and contact details confidential and will not pass this information to the sponsor, South Norfolk CCG. The local participating organisation will use this information as needed, to contact you about the research study, and make sure that relevant information about the study is recorded for your care, and to oversee the quality of the study.  The research team will keep identifiable information about you from this study for 10 years after the study has finished until 30/04/2030.  South Norfolk CCG will collect information about you for this research study from the local participating organisation. The local participating organisation will not provide any identifying information about you to South Norfolk CCG. We will use this information to enable research, which is in the public interest. Certain individuals from South Norfolk CCG, Norwich CTU and regulatory organisations may look at your medical and research records to check the accuracy of the research study. The people who analyse the information will not be able to identify you and will not be able to find out your name or contact details. |
| **If you would like any further info, please contact:** |
| Annie Blyth or Viv Maskrey, Programme Co-ordinators  Medical School, University of East Anglia, Norwich NR4 7TJ 01603 593308 or 593966 [a.blyth@uea.ac.uk](mailto:a.blyth@uea.ac.uk) /[v.maskrey@uea.ac.uk](mailto:v.maskrey@uea.ac.uk) |

**Part 2: Further information**

| **Study background** |
| --- |
| Research shows that almost 70% of care home residents experience at least one medication error on any given day.  Three recent reports and NICE guidance suggest that prescribing, monitoring and administration of medicines in care homes could be significantly improved, thus increasing residents’ quantity of life and improving use of NHS resources. Research has identified the need for one person to assume overall responsibility for the management of medicines within each care home. We propose that this role could be undertaken by a PIP working in partnership with a GP. |

| **CHIPPS Programme of Research** |
| --- |
| This study is part of CHIPPS - a large programme of research which is developing the new service described above.  The CHIPPS Programme looked at the different aspects involved, such as exactly what the service should consist of, how to train the pharmacists to be able to undertake it, how to ensure that the service is effective, safe and efficient, providing the best possible care for patients, and also how much it will cost.  The research team has been working on this for over two years now, they have tested the new service in a small study and, based on what they have learnt, are now testing the new service on a larger population. |

| **How many people are involved in this Study?** |
| --- |
| In each of the four locations (Norfolk, Yorkshire, Grampian and Belfast), we are seeking to recruit:   - 10-12 GP practices - a total of 200 – 240 residents in participating care homes. |
| **How are participants screened and recruited?** |
| - **GP practices:** All GP practices in each location will be invited to participate in the study, and from those who express an interest, the desired number will be selected - **Care homes:** Participating GP practices will select at least one of the care homes in which they provide care for the residents, and the research team will invite, on the GP’s behalf, the selected care homes. - **Participants:** All of the GPs’ patients who are resident in the selected care homes will be sent letters from the GP inviting them to participate. If the Care Home manager knows that a resident would not be able to make this decision for themselves this invitation will be sent to their relative or consultee who will respond on their behalf. If there are more than 20 eligible and willing residents they will be informed by the research team that they are not required on this occasion, and will be sincerely thanked for their interest in the study |


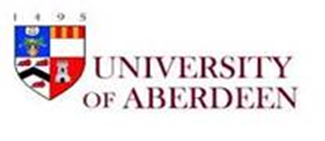

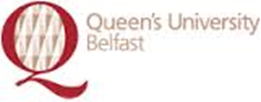

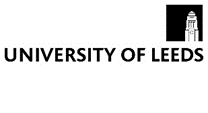

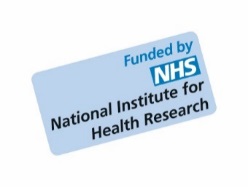


**RESIDENT - CONSENT FORM**

### Title of Study: WP6 CHIPPS Care Homes Independent Pharmacist Prescribing Study

**REC ref: TO BE ADDED**

**Name of Care home:** ____________________________

**Name of Participant:** _______________________________ **DoB Participant**: ___________

**Participant Identification Number:**

**Please initial box**

1. I confirm that I have read and understand the information sheet version number X dated xx/xx/2017 for the above study and have had the opportunity to ask questions.

2. I understand that my participation is voluntary and that I am free to withdraw at any time, without giving any reason, and without my medical care or legal rights being affected. I understand that should I withdraw then the information collected so far cannot be erased and that this information may still be used in the project analysis.

3. I understand that relevant sections of my care home notes, GP notes, and data collected in the study may be looked at by authorised individuals from the research team, regulatory authorities, or from the NHS Trust where it is relevant to my taking part in this study. I give permission for these individuals to have access to these records and to collect, store, analyse and publish information obtained from my participation in this study.

4. I understand that, with the consent of the Chief Investigator, anonymised study data may also be used by other bona fide researchers to contribute to future research. I understand that my personal details will be kept confidential, and I will not be identifiable in any public output, or data shared outside the immediate research team.

5. I agree to my GP or other health and social care professionals being informed of my participation in this study.

6. I agree to my contact details and a copy of this consent form being held securely and confidentially by CHIPPS coordinating centre/Norwich Clinical Trials Unit.

7. I am happy to be contacted about participating in additional research interviews which are part of this study.

8. I agree to take part in the above study.

9. I agree to continue participating in the study if I lose capacity before the end of the study.

_____________________ ______________ ________________________­­­_­­­_

# Name of Participant Date Signature

_______________________ ______________ __________________________

Name of Person taking consent Date Signature


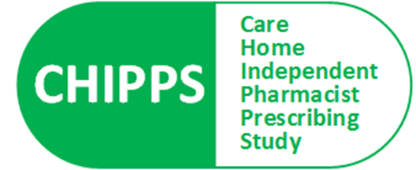


**CHIPPS WP6 Letter Consultee first letter**

**(Version 1, 8^th^ August 2017)**

**Care Home**: ________________________ Date: ________________

Dear [INSERT NAME]

**Re:** ________________________________________________________________

We are writing to you about a study which is taking place in this care home. We would like to ask you to be a Consultee, on behalf of your friend/relative. A Consultee is someone who gives advice on behalf of a relative or friend.

Patients of your relative/friend’s GP who live in this care home are being invited to take part in a study of a new service. This may involve a specially qualified pharmacist prescriber working in partnership with your relative/friend’s GP and taking responsibility for your relative/friend’s prescriptions. Please see the attached leaflet for more information.

We are writing to you as your relative/friend is not able to decide for themselves whether they would like to take part or not.

We are asking you to advise us about whether they would like to join the study.

***Would your relative/friend have agreed to take part if they had been able to decide for themselves?***

We would like you to consider what you know of their wishes and feelings, and to consider their interests. Please let us know of any advance decisions they may have made about participating in research.

If you are unsure about taking on the role of Consultee you may seek independent advice*. We will understand if you do not want to take on this responsibility.

If you decide your relative/friend would like to take part please read and sign the Consultee declaration enclosed with this letter and return it to us in the envelope provided.

If you decide that your relative/friend would not wish to take part it will not affect the standard of care they receive in any way.

If you have any questions, or you would like to talk to the researchers, please contact them using the details in the attached information sheet. Alternatively if you would like to speak to a member of staff at the care home about the study, please speak to me [XX Name, Care Home Manager].

We would be most grateful if you could respond within the next two weeks.

Yours sincerely,

[XX Name] Care Home Manager

* Further information is available in the Department of Health’s ‘Guidance on nominating a consultee for research involving adults who lack capacity to consent’.

<http://webarchive.nationalarchives.gov.uk/20130107105354/http://www.dh.gov.uk/prod_consum_dh/groups/dh_digitalassets/@dh/@en/documents/digitalasset/dh_083133.pdf>

This is also available from the research team, please ask if you would like a copy.


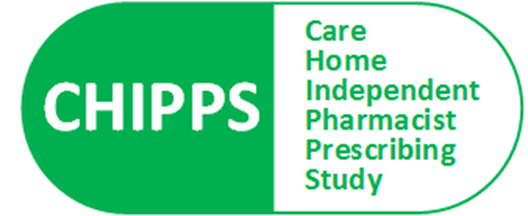

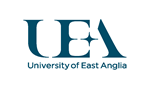


**We are inviting your relative/friend to take part in our project**

**research project**

**CHIPPS**

**Care Homes Independent Pharmacist Prescribing Study**

# Information sheet for Participant’s Consultee

This leaflet tells you about our project. This is a project which involves a qualified pharmacist working in care homes in partnership with your friend/relative’s GP.

**Part 1: Essential information**

The pharmacist has had extra training to be able to prescribe medicines. They are called Pharmacist Independent prescribers (PIPs). Nationally PIPs are increasingly working within GP practices to help patients get the best from their medicines. During the project the pharmacist will share responsibility with your friend/relative’s GP for the prescribing of their medicines.

The project is NOT testing any new pharmacist activity or experimental drugs; nor does it involve any extra blood tests or hospital visits, in addition to those that your friend/relative may already receive. We want to be sure you understand the project before you advise on your friend/relative’s wishes. Feel free to ask any questions.

**We are inviting people who live in this care home, and take at least 1 medicine regularly, to take part in our project.**

Your friend/relative does not have to take part and they may withdraw from the project at any time without stating a reason. This will not affect their legal rights, medical treatment or social care in any way.

We want to see if a PIP, working in partnership with their GP, can help patients to get the best possible benefit from their medicines, and also keep an eye on their symptoms. In particular, we are interested to see whether this prevents people from having problems with their medicines and if it is a more effective way of managing medicines for people living in care homes.

**Here are some answers to questions you may have:**

| **What happens if my friend/relative takes part?** |
| --- |
| Your friend/relative will be put at random into one of two groups (as if by ‘the toss of a coin’). The people who are put into group 1 will receive the new service; those in group 2 will not receive the new service, and their care will continue as normal. Your friend/relative will not be told what group they are in.  For those in group 1, the PIP, who is part of your friend/relative’s GP practice’s staff, will share responsibility for their medicines, including prescribing for the next 6 months. The PIP may arrange to see or telephone you to discuss how your friend/relative is and to talk to you about their medicines and any problems they have with them. It is ok if you don’t know about your friend/relative’s medicines as the PIP will be working with the staff here at the care home to help your friend/relative get the best from their medicines.  Occasionally, the PIP may suggest that changes are made to the medicines. As they are prescribers, the PIP will be able to organise this and will make a record of any changes on your friend/relative’s medical records at the GP practice so that everyone involved in their care knows about the changes.  Whichever group your friend/relative is in, the researcher will ask your permission to look at your friend/relative’s records held in the care home and the GP practice, and record their medical history, list medicines they have been prescribed and the number of times they have seen the GP or other health care providers, We will also ask the Care Home staff to tell us how your friend/relative is doing, using standard questionnaires.  If your friend/relative is suddenly unwell they will continue to receive their normal service from their GP. |

| **Your role?** |
| --- |
| We are asking you to advise us about whether they would like to join the study. Would your friend/relative have agreed to take part if they had been able to decide for themselves?  We ask you to consider what you know of their wishes and feelings, and to consider their interests. Please let us know of any advance decisions they may have made about participating in research.  If you are unsure about taking the role of consultee you may seek independent advice. We will understand if you do not want to take on this responsibility.  If you do think your friend/relative would like to take part, then please complete the enclosed ‘**CONSULTEE – ADVICE FORM’**.  The ‘**ADVICE FORM’** asks your permission to look at your friend/relative’s records held in the care home and the GP practice. This is to record the medicines they have been prescribed and their health care. |

| **What possible benefits are there to taking part?** |
| --- |
| A PIP, who has been specially trained to work with your friend/relative’s GP, may help your friend/relative get the best from their medicines; this is something pharmacists are experienced in and are used to doing. |

| **What disadvantages are there?** |
| --- |
| We do not think there are any disadvantages. However, you may not want to have the PIP prescribing medicines for your friend/relative, in which case you will probably not want them to take part. |
| **What happens when the project comes to an end?** |
| At the end of the six month project it will be up to the individual care home, in discussion with the GP and pharmacist, whether they continue to provide the service after the research is completed.  This decision will be made taking into account the findings of the study. These findings will also inform future NHS recommendations for the role of pharmacists in care homes. We aim to publish the results of this project both locally and nationally. These reports will not include names or other personal details that would allow you or your friend/relative to be identified. |

| **Confidentiality – Who will know information about my relative/friend from this project?** |
| --- |
| All those involved will treat personal information in absolute confidence. Information about the medicines your friend/relative are taking, the number of times that they see the PIP, GP and other health care professionals will be passed only to the research staff working on the project. |

| **Who is organising and funding the study?** |
| --- |
| The study is funded by the NHS through the National Institute of Health Research [RP-PG-0613-20007], and is being co-ordinated by researchers at the University of East Anglia, with research partners at the Universities of Aberdeen, Leeds and Queen’s University Belfast. |
| **What if there’s a problem?** |
| If you have any concerns about the study, you should raise them first directly with the researcher (see details below).  If you have further concerns please contact the Sponsor’s representative Clare Symms, Research Governance Manager, Norfolk & Suffolk Primary & Community Care Research Office, Hosted by South Norfolk CCG, Lakeside 400, Old Chapel Way, Broadland Business Park, Thorpe St Andrew, Norwich, NR7 0WG 01603 257020 |

| **Who has assessed the ethical implications of the research?** |
| --- |
| The research has been approved by East of England NHS Research Ethics Committee |

| **What will happen to the data collected?** |
| --- |
| South Norfolk CCG is the sponsor for this study based in the United Kingdom. We will be using information from you and your medical records in order to undertake this study and will act as the data controller for this study. This means that we are responsible for looking after your information and using it properly.  Your rights to access, change or move your information are limited, as we need to manage your information in specific ways in order for the research to be reliable and accurate. If you withdraw from the study, we will keep the information about you that we have already obtained. To safeguard your rights, we will use the minimum personally-identifiable information possible.  You can find out more about how we use your information at: <http://www.southnorfolkccg.nhs.uk/public-information/fair-processing-notice>  The local participating organisation will collect information from you and your medical records for this research study in accordance with our instructions.  The local participating organisation will keep your name and contact details confidential and will not pass this information to the sponsor, South Norfolk CCG. The local participating organisation will use this information as needed, to contact you about the research study, and make sure that relevant information about the study is recorded for your care, and to oversee the quality of the study.  The research team will keep identifiable information about you from this study for 10 years after the study has finished until 30/04/2030.  South Norfolk CCG will collect information about you for this research study from the local participating organisation. The local participating organisation will not provide any identifying information about you to South Norfolk CCG. We will use this information to enable research, which is in the public interest. Certain individuals from South Norfolk CCG, Norwich CTU and regulatory organisations may look at your medical and research records to check the accuracy of the research study. The people who analyse the information will not be able to identify you and will not be able to find out your name or contact details. |

| **If you would like any further info, please contact:** |
| --- |
| Annie Blyth or Viv Maskrey, Programme Co-ordinators  Medical School, University of East Anglia, Norwich NR4 7TJ Phone: 01603 593308 or 593966  Email: [a.blyth@uea.ac.uk](mailto:a.blyth@uea.ac.uk) /[v.maskrey@uea.ac.uk](mailto:v.maskrey@uea.ac.uk) |


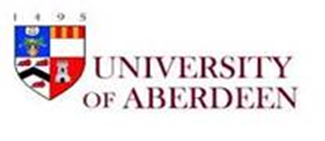

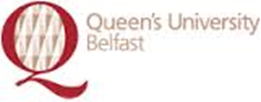

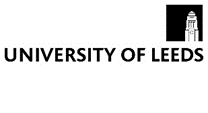

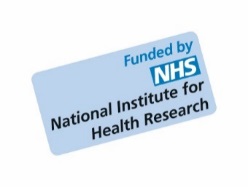


**Part 2: Further study information**

| **Study background** |
| --- |
| Research shows that almost 70% of care home residents experience at least one medication error on any given day.  Three recent reports and NICE guidance suggest that prescribing, monitoring and administration of medicines in care homes could be significantly improved, thus increasing residents’ quantity of life and improving use of NHS resources. Research has identified the need for one person to assume overall responsibility for the management of medicines within each care home. We propose that this role could be undertaken by a PIP working in partnership with a GP. |

| **CHIPPS Programme of Research** |
| --- |
| This study is part of CHIPPS - a large programme of research which is developing the new service described above.  The CHIPPS Programme looked at the different aspects involved, such as exactly what the service should consist of, how to train the pharmacists to be able to undertake it, how to ensure that the service is effective, safe and efficient, providing the best possible care for patients, and also how much it will cost.  The research team has been working on this for over two years now, they have tested the new service in a small study and, based on what they have learnt, are now testing the new service on a larger population. |

| **How many people are involved in this Study?** |
| --- |
| In each of the four locations (Norfolk, Yorkshire, Grampian and Belfast), we are seeking to recruit:   - 10-12 GP practices - a total of 200 – 240 residents in participating care homes. |
| **How are participants screened and recruited?** |
| - **GP practices:** All GP practices in each location will be invited to participate in the study, and from those who express an interest, the desired number will be selected - **Care homes:** Participating GP practices will select at least one of the care homes in which they provide care for the residents, and the research team will invite, on the GP’s behalf, the selected care homes. - **Participants:** All of the GPs’ patients who are resident in the selected care homes will be sent letters from the GP inviting them to participate. If the Care Home manager knows that a resident would not be able to make this decision for themselves, this invitation will be sent to their relative or consultee who will respond on their behalf. If there are more than 20 eligible and willing residents they will be informed by the research team that they are not required on this occasion, and will be sincerely thanked for their interest in the study |

**CONSULTEE – ADVICE FORM**

**(Version 1, 08.08.2017)**

### Title of Study: CHIPPS Care Homes Independent Pharmacist Prescribing Study

**REC ref: XX/XX/XXXX**

**Name of Researcher**:

**Name of Participant: d.o.b:**

**Name of Care Home: _______________________________**

**Participant Identification Number:**

**Please initial box**

1. I have read the Participant Information Sheet (version x, date xx/xx/2017) and the nature and purpose of the research project is clear to me. I have been given the opportunity to think about the study, ask questions and those questions have been answered satisfactorily.

2. I understand that the resident’s participation is voluntary. They can withdraw from the study at any stage, without giving reasons and this will not affect their legal rights, medical or social care, now or in the future. I understand that should they withdraw then the information collected so far cannot be erased and that this information may still be used in the project analysis.

3. I understand that relevant sections of their care home notes, GP notes, and data collected in the study may be looked at by authorised individuals from the research team, regulatory authorities, or from the NHS Trust where it is relevant to their taking part in this study. I give permission for these individuals to have access to these records and to collect, store, analyse and publish information obtained from their participation in this study. I understand that their personal details will be kept confidential.

4. I agree to their GP or other health and social care professionals being informed of their participation in this study.

5. I agree to my contact details and a copy of this consent form being held securely and confidentially by CHIPPS coordinating centre/Norwich Clinical Trials Unit.

6. I am happy to be contacted about participating in one additional research interview which is part of this study.

7. In my opinion, the resident would have no objection to taking part in the above study.

**_____________________ ______________ ________________________**

# Name of Consultee Date Signature

**_____________________**

# Relationship of Consultee to Resident

**_______________________ ______________ __________________________**

Name of Researcher Date Signature

4 copies: 1 for participant, 1 for location file (original), 1 for care home study file, 1 for Norwich CTU Trial Office
