# Supplementary material for: Protocol for a cluster randomised controlled trial to determine the effectiveness and cost-effectiveness of independent pharmacist prescribing in care homes: the CHIPPS study
Source: Trials. 2020 Jan 21;21:103. doi: 10.1186/s13063-019-3827-0 (PMC6975047; doi:10.1186/s13063-019-3827-0)
Supplement: Supplementary file 3 — Additional file 3. Capacity assessment for residents. [file 13063_2019_3827_MOESM3_ESM.doc]

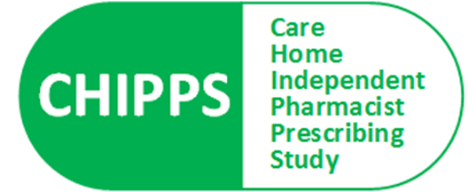

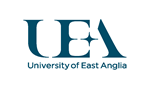


**Capacity assessment for residents**

Resident name: Date of birth:

Care home name:

Researcher to work through the Participant information sheet before asking the following questions. Researcher to complete this section:

|  | Question to the researcher | Yes | No |
| --- | --- | --- | --- |
| 0 | Is it apparent that the resident lacks capacity? |  |  |

**If yes do not proceed with questions 1-3**

|  | **Question to ask of the resident** | **Necessary reply** | **Appropriate reply** | |
| --- | --- | --- | --- | --- |
|  |  |  | Yes | No |
| 1 | Can you tell us what the study is about? | Mention of pharmacist working with their GP and prescribing medicines |  |  |
| 2 | If you take part in the study, what will happen to you? | Mention pharmacist may visit them at the care home |  |  |
| 3 | Does everyone need to take part? Can I refuse without it affecting my care or my relationship with my GP? | I can refuse. No, there would be no consequences, care and support would not alter. |  |  |
| Resident able to provide informed consent? Only tick “yes” if all questions 1-3 provided an appropriate reply (marked “yes”) | | |  |  |

Researchers name: ……………………………………………….

(print)

Researcher signature: ……………………………………………

Date: ……………………………………………………………………

Notes on process (note if participant lost interest or was upset etc.):
